# Associated injuries in patients with facial fractures: a review of 604 patients

**DOI:** 10.11604/pamj.2013.16.119.3379

**Published:** 2013-11-27

**Authors:** Rasmané Béogo, Patrick Dakouré, Léon Blaise Savadogo, Antoine Toua Coulibaly, Kampadilemba Ouoba

**Affiliations:** 1 Department of Oral and Maxillofacial Surgery, CHU Sanou Souro, Burkina Faso; 2 Department of Orthopaedics and Trauma surgery, CHU Sanou Souro, Burkina Faso; 3 Department of Epidemiology and Public Health, CHU Sanou Souro, Burkina Faso; 4 Department of OtoRhino Laryngology, CHU Yalgado Ouédraogo, Burkina Faso

**Keywords:** Face, Facial fracture, Associated injury

## Abstract

Facial fractures may be associated with concomitant lesions of other parts of body with some of these injuries being life-threatening. This retrospective study reports the types of associated injury and the factors influencing their occurrence, in patients with facial fractures. In 18.2% of 604 patients, one associated injury at least was recorded. The most common associated injury was cranial trauma (9.9%), followed by limbs fractures (9.1%), chest trauma (2%), spine injury (0.5%) and eye ball rupture (0.5%). A poly trauma was recorded in 3.2% of the patients who had sustained a cerebral trauma, a spinal injury or a thoracic trauma. Death occurred in two patients (0.3%) who had respectively a spinal injury and a chest trauma. The occurrence of associated injuries correlated significantly with the fracture type with solitary mandibular fracture being a significant predictor of associated injuries. Although not statistically significant, multiple facial fractures and violence were more associated with concomitant injuries. The findings of this study recall the need for initial full examination of the trauma patients particularly victims of violence, patients presenting with multiple facial fractures or single facial bone fracture involving the mandible, the trauma patients? multidisciplinary management as well as trauma prevention.

## Introduction

Facial fractures patients may experience a variety of injuries of other regions of the body. These associated injuries (AIs) worsen the facial trauma prognostic as some of them may result in functional disabilities or even death. Their risk of occurrence and types vary according to some factors such as the mechanism of the facial fracture [1]. Most of the studies on facial fractures AIs are from developed countries where facial traumas are mainly caused by interpersonal violence [1, 2]. Reports from developing countries where the leading aetiology is road traffic crashes [3, 4] are scarce. The aim of this study was to determine the types and occurrence of AIs and their influencing factors, in patients with facial fractures. This knowledge may assist in appropriate management of the facial trauma patients.

## Methods

A retrospective descriptive study was carried out between 2001 and 2010 on the concomitant injuries in 604 patients with facial fractures at CHU Souro Sanou, a referral hospital in Burkina Faso. AI was defined as any extra facial injury excluding brain commotion and wounds i.e. intracranial, vascular, thoracic or abdominal organs injuries, fractures other than those of the face. The collected data included the patient's age and gender, the trauma aetiology, the facial fracture site, the AI type and site. The diagnosis of facial fractures and AIs was based on clinical and radiological findings. The Chi Square test was used to compare proportions; the difference was significant when p value <0.05. The study didn't require ethical approval according to the IRB standards of the institution in which it was carried out.

## Results

### Patients and facial fracture characteristics

Facial fractures patients? age ranged from 1 to 75 years (mean, 30.3 years). Their incidence peak (57.8%) was observed from 20 to 39 years. The male to female ratio was 8:1. Road traffic accidents were by far the leading aetiology and involved in near 70% of the cases, two-wheel vehicles. Out of the victims of fall, 39 fell from trees. Six cases violence involved firearms. Other aetiologies comprised falls at home (8 patients), sport accidents (7), game (2), tyre blast (2), and animal attack (1) (Table 1).

**Table 1:** Facial fracture patients’ distribution according to gender, age, and fracture aetiology and site

	n	%
**Gender**		
Male	537	88.9
Female	67	21.1
**Age**		
0 – 19 years	118	19.5
20 – 39 years	349	57.8
>40 years	137	22.7
**Aetiology of facial fracture**		
Road traffic accidents	483	80
Violence	60	9.8
Fall from height	41	6.8
Other	21	3.4
**Site of facial fracture**		
Mandible	374	61.9
Zygomatic complex	253	41.9
Le Fort fracture	148	24.5
Nasofrontoorbital ethmoid complex	22	3.6

**Table 2:** Distribution of patients with associated injuries according to gender, age, and fracture aetiology and site

	n	%	p
**Gender**			0.7
Male	99	18.4	
Female	11	16.4	
**Age**			0.1
0 – 19 years	118	20	
20 – 39 years	349	57	
>40 years	137	33	
**Aetiology of facial fracture**			0.6
Road traffic accidents	87	18	
Violence	13	21.6	
Fall from height	8	16.3	
Other	2	9.5	
**Number of facial bones**			0.14
One facial bone	75	16.8	
Multiple facial bones	35	22	
**Type of facial bone**			0.02
Mandible	53	21.4	
Zygomatic complex	16	11.1	
Le Fort fracture	6	10.9	

Facial fracture involved most commonly the mandible, followed by the zygomatic complex and the maxilla. The maxilla was the most commonly involved in combined facial fractures (62.9% versus 43.5% and 34% for respectively the zygomatic complex and the mandible). The nasofrontoorbital ethmoid complex fracture was always associated with another facial fracture.

### Types and frequency of AIs

**Figure 1:**
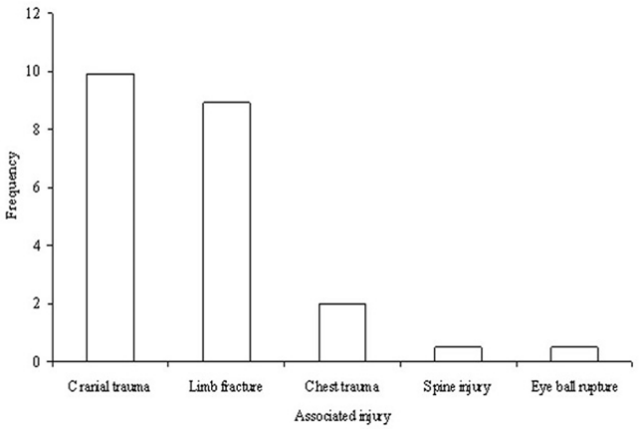
Types and frequency of associated injuries

Out of 604 patients with facial fractures, 110 sustained at least one AI, giving a frequency of 18.2% of AIs patients. These 110 patients had 132 AIs (90 had one AI, 18 had two AIs and two had three AIs). As shown in Figure 1, the most common AI was cranial trauma (9.9%, 60/604) followed by limb fractures (9.1%, 55/604). Poly trauma was recorded in 2.3% (14/604) of the patients who presented brain injury with a GCS < 8 (10 patients), cervical spine injury (3 patients), and chest trauma with respiratory distress (1 patient). Death was recorded in two patients who presented respectively a chest trauma and a cervical spine injury, giving a mortality rate of 0.3% (Figure 1).

### Factors of occurrence AIs

AIs occurrence was not statistically associated with age and gender. AIs occurred more often in violence victims and patients presenting with multiple facial fractures although not significantly. In single facial bone fractures, AIs were significantly more observed in mandibular fracture than in the Le Fort and zygomatic fractures.

## Discussion

In the literature, reports on facial fractures and their concomitant injuries are mostly focused on a specific type of facial fracture such as those of the mandible and its related AIs or on a particular injury such as spine injury. Studies dealing extensively with all facial fractures and all AIs like this study are scarce. AIs are reported in these studies with a wide variation of incidence ranging from 8.3% to 99.3% [1, 4–8]. This variation can be explained by lack of standard definition of AIs, variations in aetiologies of facial fractures, study populations? characteristics and reporting bias [7, 9]. In a study reporting AIs rate reaching up to 99.3%, head lacerations are defined as concomitant injury [10].

The rates of AIs (18.2%), polytrauma (2.3%) and mortality (0.3%) in this study may suggest mild trauma resulting from low velocity. However, there could be an underestimation of theses rates as most of severely traumatized patients with maxillofacial fractures and associated injuries may die prior to any accurate assessment of their injuries particularly in setting of absence of proper resuscitation. According to Down et al, 50% of these patients die before or soon after arrival to hospital [11]. More over, in surviving patients, some AIs may be misdiagnosed in setting of lack of full and accurate diagnosis facilities. Observation of cranial injuries and limb fractures as being by far the most common AIs in this study is also reported in several [1, 2, 4–6, 8] whilst other authors report cervical spine injuries [12, 13]. One of the reasons of the lower incidence of cervical spine injury in this study may be the fact that it is not a routine in our practice to perform computed tomography of cervical spine in facial fractures patients. Despite a young and male predominance in the patients with facial fractures, there is no significant difference in age and gender with regard to the AIs occurrence in this study. Thorén even reports AIs more prevalent in older than in younger patients [1].

Compared to road traffic accidents, violence tends to be associated with isolated fractures and a lower frequency of concomitant injuries as the aggressor commonly targets the prominent points on the face [14, 15]. In motor vehicles accidents, patients are more at risk of multiple facial fractures and AIs as they are exposed to a trauma with high velocity and random distribution [8, 16]. Contrasting findings in this series are likely due to differences in road traffic accidents and interpersonal violence characteristics in this study setting. The leading rank of road traffic accidents in the aetiologies of facial fractures is reported in most of the studies from developing countries [3, 4]. Violence deserves a special awareness in this study as the most common aetiology of facial fractures after traffic accidents and the greatest provider of AIs. It has emerged as the leading aetiology of facial fractures in series from developed countries [1, 2]. Occurrence of AIs more in multiple facial fractures than in single facial fracture although not statistically significant in this study, is intuitively expected. Trauma able to fracture multiple facial bones is also likely to have velocity and distribution to cause injuries of other parts of the body than the facial skeleton [8]. In single facial fractures, occurrence of AIs more in the mandible than in the maxilla and zygomatic complex in this study may be explained by patient survival. Several studies report Le Fort fracture to be the most common facial fracture in severely injured patients [2, 9]. Such patients can hardly survive in this study setting as patient's proper transportation and resuscitation facilities are lacking. Plaisier et al, in a series of 661 facial fracture patients, report non-survivors to have more likely midfacial fractures [17]. Mandible fractures could result more from trauma with enough velocity to result in the fracture of this thick bone and concomitant injuries but non-lethal injuries such as limb fracture.

## Conclusion

Concomitant injuries are not uncommon in facial fracture patients with some of them being lethal. Initial full examination of the patients particularly victims of violence, patients presenting with multiple facial fractures or single facial bone fracture involving the mandible, is recommended as well as multidisciplinary management and trauma prevention.
